# Fluoropolymers as Unique and Irreplaceable Materials: Challenges and Future Trends in These Specific Per or Poly-Fluoroalkyl Substances *

**DOI:** 10.3390/molecules28227564

**Published:** 2023-11-13

**Authors:** Bruno Améduri

**Affiliations:** Institute Charles Gerhardt, University Montpellier, CNRS, ENSCM, 34293 Montpellier, France; bruno.ameduri@enscm.fr

**Keywords:** end of life, fluoropolymers, per- and polyfluoroalkyl substances (PFASs), polymerization aids, recycling, regulators, surfactants

## Abstract

In contrast to some low-molar-mass per- and polyfluoroalkyl substances (PFASs), which are well established to be toxic, persistent, bioaccumulative, and mobile, fluoropolymers (FPs) are water-insoluble, safe, bioinert, and durable. These niche high-performance polymers fulfil the 13 polymer-of-low-concern (PLC) criteria in their recommended conditions of use. In addition, more recent innovations (e.g., the use of non-fluorinated surfactants in aqueous radical (co)polymerization of fluoroalkenes) from industrial manufacturers of FPs are highlighted. This review also aims to show how these specialty polymers endowed with outstanding properties are essential (even irreplaceable, since hydrocarbon polymer alternatives used in similar conditions fail) for our daily life (electronics, energy, optics, internet of things, transportation, etc.) and constitute a special family *separate* from other “conventional” C_1_–C_10_ PFASs found everywhere on Earth and its oceans. Furthermore, some information reports on their recycling (e.g., the unzipping depolymerization of polytetrafluoroethylene, PTFE, into TFE), end-of-life FPs, and their risk assessment, circular economy, and regulations. Various studies are devoted to environments involving FPs, though they present a niche volume (with a yearly production of 330,300 t) compared to all plastics (with 460 million t). Complementary to other reviews on PFASs, which lack of such above data, this review presents both fundamental and applied strategies as evidenced by major FP producers.

## 1. Introduction

According to the Organization for Economic Co-operation and Development (OECD) [1,2], perfluoroalkyl (and polyfluoroalkyl) substances (PFASs) are artificial/anthropogenic products that contain multiple C–F bonds [3,4,5,6,7,8,9]. Because of their unique hydrophobic/lipophobic physicochemical properties, this specific group of chemicals displays quite different physical, chemical, and biological properties [5,6,7,8,9] than other hydrocarbon compounds and have been involved in many applications in different areas [5,6,7,8,9,10]. The OECD [1] has reported more than 4700 PFAS molecules and, according to the US Environment Protection Agency [3,11], this number reaches more than 12,000 compounds [12]. However, PFAS molecules, especially those of low molar mass, have been found in many soils, waters, seas, and oceans as reported by a plethora of articles and reviews [4,5,6,7,8,9,10]. Thanks to the high electronegativity and small radii of the fluorine atom that confers a short and quite stable C-F bond, fluorinated products have remarkable chemical inertness and thermal stability (the dissociation energy of this C-F bond is worth ca. 120 kcal·mol^−1^ [7,9]). However, such characteristics further induce some limitations, since PFASs are known to be water-soluble, mobile, toxic, persistent. and bioaccumulable [9,12,13,14]. Since 2000, many regulations have been proposed, modified, and multiplied [5,6,7,8,10,12,13,14,15,16,17]. Various fluorinated compounds have been banned for several years and continuous procedures from competent authorities still maintain the pressure to impose drastic legislations/regulations [16].

Thomas et al. [17] comprehensively reported the global situation on the regulation of PFASs in both the scientific and legislative communities, and analyzed the different regulatory actions launched both in Europe and in the USA (including federal- and state-level initiatives). These authors supplied an exhaustive list of different dossiers of legislation and carefully reported how regulators have approached the concerns regarding PFASs from different perspectives. Last March 2023, China added PFOA and PFOS to its list of New Pollutants for Priority Management to modify their production, use, import, and export [18]. Presently, 152 countries have ratified the Stockholm Convention, which puts in place the elimination and restriction of listed PFASs with exemptions for specific uses such as hard metal plating and firefighting foams (AFFFs). As of 5 April 2023, Israel, Haiti, the USA, Malaysia, and Brunei Darussalam have not ratified the convention. Although ratified, there is no indication that a number of the member nations have made efforts to regulate PFASs via other independent mechanisms [17].

This review aims first at making a distinction between various classes of PFASs, showing major issues from low-molar-mass ones in contrast to the high-molar-mass fluoropolymers, which display specific advantages (safety, bioinertness, non-toxicity, and fulfilling the 13 polymer-of-low-concern (PLC) criteria in their conditions of use).

In addition, advanced technologies from industrial manufacturers of FPs have led to recent innovations such as the use of non-fluorinated surfactants in the aqueous radical (co)polymerization of fluoroalkenes to yield FPs with similar performances. These above innovations have unprecedentedly been reported before. This review also aims at showing how these specialty polymers endowed with outstanding properties are essential (even irreplaceable) for our daily life (in electronics, energy, optics, internet of things, transportation, etc.) and constitute a special family that is *separate* from the other “conventional” C_1_–C_14_ PFASs found everywhere on Earth and its oceans. Furthermore, some information reports on their recycling (e.g., the unzipping depolymerization of polytetrafluoroethylene, PTFE, into TFE), end-of-life FPs, and their risk assessment and circular economy. Various studies are devoted to environments involving FPs, though they represent a niche volume. Complementary to articles on PFASs, which lack the information above, this review gathers both fundamental and applied strategies as evidenced by major FP producers. Various features are still attracting huge interest from both academic and industrial players.

## 2. History of PFASs

**Figure 1:**
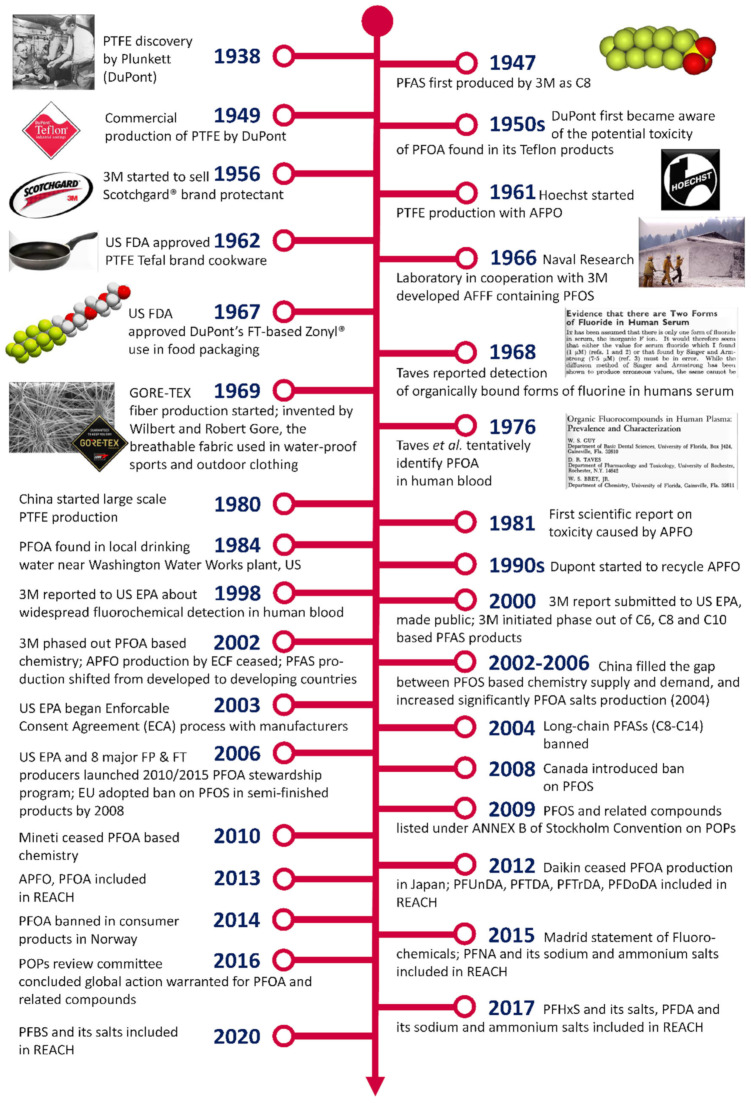
Historical scheme of the fluorochemicals, from the discovery of polytetrafluoroethylene (PTFE) to REACH regulations (Reprinted with permission from Ref. [9]. Copyright 2022 Royal Society of Chemistry) (APFO, ECF, EPA, FT, PFBS, PFCA, PFNA, PFOA, PFOS, POP, and REACH stand for ammonium perfluorooctanoate, electrochemical fluorination, Environment Protection Agency, fluorotelomer, perfluorobutanesulfonate, perfluorocarboxylic acid, perfluorononanoic acid, perfluorooctanoic acid, perfluorooctanesulfonic acid, persistant organic pollutants, and Registration, Evaluation, Authorisation and Restriction of Chemicals, respectively.

**Figure 2:**
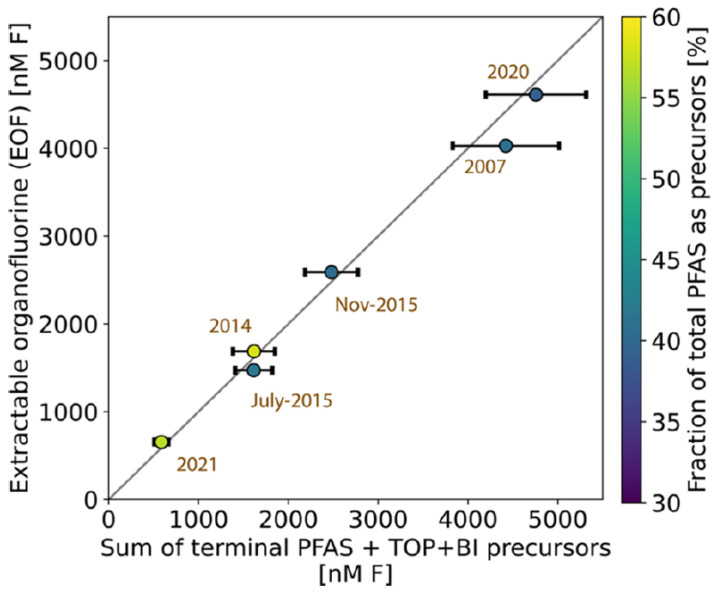
Mass budget for PFASs in groundwater below the fire training area at Joint Base Cape Cod, USA: Comparison of detected extractable organofluorine (EOF) and PFAS concentrations. The *x*-axis represents the sum of terminal PFAS and oxidizable precursors inferred from the total oxidizable precursor (TOP) assay using Bayesian inference. The sum of differences from duplicate extracts for targeted PFAS (9%; *n* = 1) and the standard deviation of inferred precursor concentrations are shown by *x*-axis error bars. Circles are shaded in purple according to the fraction of EOF explained by oxidizable precursors. The one-tone line (gray) is used to compare the sum of terminal PFASs and precursors measured using targeted analysis and the TOP inference with EOF. Reprinted with permission from Ref. [24].

Fluorinated polymers have been known for eighty-five years [14] and a brief historic sketch showing their extraordinary evolution is depicted in Figure 1, which includes fluorinated gases (discovered in the 1920s), intermediates, monomers, oligomers, and polymers, as well key methods to supply them either via electrochemical fluorination [9] or telomerization [19]. Among them, surface-active products (surfactants) are specific amphiphilic PFASs that display much lower surface tension and surface energy than hydrocarbon surfactants [9,20]. They are currently used in more than 200 applications, such as aqueous firefighting/film-forming foams [20,21,22], food packaging, cosmetics, photoacid generators [9,10,20], paints, semiconductors, in electroplating [22], and as polymerization aids in the emulsion radical polymerization of fluorinated alkenes [9,22]. There are many examples related to PFASs including their production sites [12] or storage areas such as military bases equipped with extinguishers [23,24] and airports [25,26], where many PFASs have been found. Ruyle et al. [24] analyzed groundwater samples from a fire training area between September 2007 and July 2021 (six time points; Figure 2). All groundwater measurements were from samples collected from the same well to control the hydrological variability. The samples represent concentrations in the upper 2–3 m of groundwater. These authors directly compared the PFAS concentrations to those of samples collected in 2007. Their analyses were achieved in order to supply quantitative and qualitative information from complementary techniques such as liquid chromatography–tandem mass spectrometry (LC-MS/MS), combustion ion chromatography, high-resolution mass spectrometry, and a comparison of measured and library MS/MS spectra.

## 3. Issues on Mobility, Persistency, Toxicity, and Bioaccumulation of PFASs

As a matter of fact, major concerns arise from their high water solubility [27] and thus mobility, as well as their chemical stability, toxicity, persistence [26,28,29,30], and bioaccumulation in the environment, in the food chain, and in humans [5,6,10,12,23,28,29,30,31]. Growing attention has been paid to PFASs as a specific chemical class [4,7,31] because of their adverse health effects, modes of action, and physical and biochemical properties [4], mainly linked to their too-stable small perfluorinated chain that cannot degrade or be metabolized.

Actually, modern powerful analytical apparatus and machines are able to detect very low chemical concentrations (as low as several tenth of ppb [32] (or even hundreds of ppt); knowing that one ppb is a drop of water in an Olympic-size swimming pool!).

Globally, environmental officials from five member states [16] launched actions to push regulatory agencies (as Registration, Evaluation, Authorisation and Restriction of Chemicals: REACH) and consequently the EU to restrict PFASs (including FPs) with some plans to ban all PFASs by 2030 [33]. In addition, more than a dozen states in the USA have been also influencing authorities to ban PFASs in the coming years (Maine and Minnesota have already started). Consequently, synthesizing alternatives to face perfluorooctanoic acid (PFOA) and perfluorooctane sulfonic acid (PFOS) issues (since 2006, PFOA and PFOS, their salts, and similar compounds have been banned in many countries) led to a large amount of research that has been well summarized in various reviews [21] or book chapters [22,34] (though China is still currently using PFOA as polymerization aid as well as other PFASs containing shorter chain lengths). On the other hand, Chemours and Dyneon industrially produced hexafluoropropylene oxide dimer ammonium carboxylate (Gen^®^X or HFPO-DA) and 4,8-dioxa-3H-perfluorononanoate (Adona^®^), respectively, enabling FPs to be yielded, the quality and performances of which were similar to those of the FPs obtained in the presence of PFOA or PFOS. However, several years later, these surfactants had also been detected in soils and rivers, probably because of their persistency and mobility issues [9,34,35].

## 4. Different Categories of PFASs

**Figure 3:**
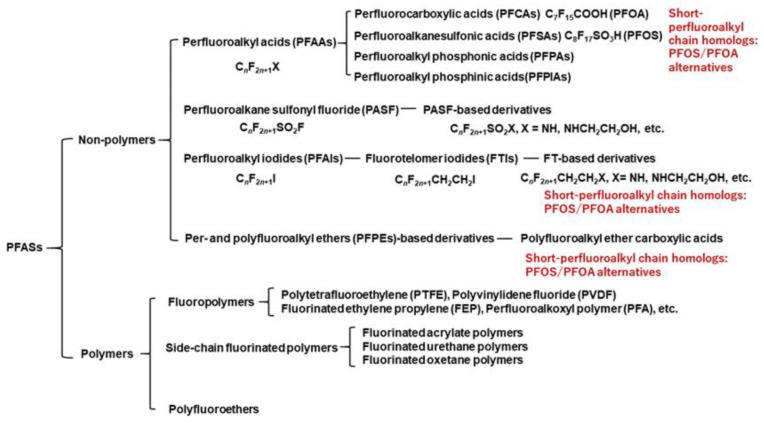
General classification of PFASs according to the Organization for Economic Co-operation and Development (OECD) [1].

PFASs have been categorized into two distinct families of fluorinated compounds [4,7,27,36], the major differences being their molar mass (Figure 3) and dispersity for their separate uses.

### 4.1. Non-Polymeric PFASs

#### 4.1.1. Introduction

**Scheme 1:**
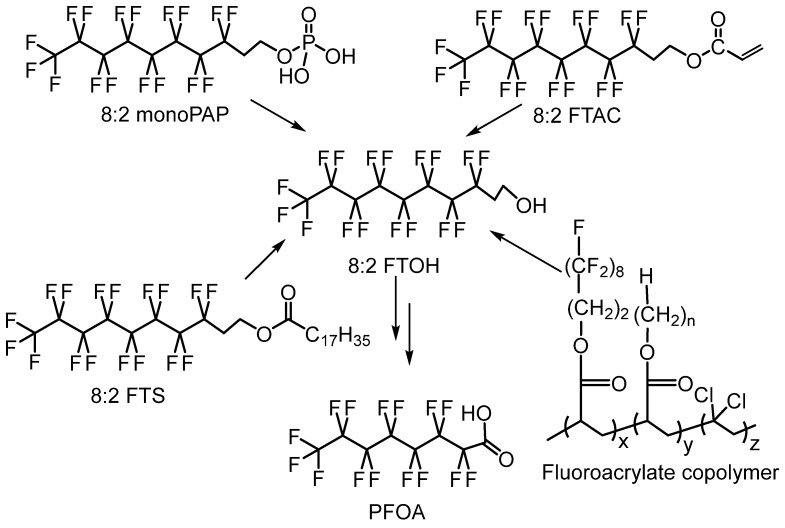
Illustration of microbial degradation of poly(fluoroacrylate) that generates 8:2 fluorotelomer derivatives. Adapted with permission from Ref. [43]. Copyright 2013 Elsevier.

Low-molar-mass PFASs (<1000 g·mol^−1^), including PFOA, PFOS, and fluorinated telomers (Scheme 1) [9,19], have contaminated many places on Earth and its oceans or decompose in the stratosphere, leading to trifluoroacetic acid (which is extremely difficult to separate from water) and trifluoroacetate [37], polluting waters and soils and thus bringing negative impacts. These compounds also cross the cellular membranes of animals and human beings [1,32,38].

#### 4.1.2. Strategies to Eliminate Low-Molar-Mass PFASs

Beside the thermal (low [39] or high [40,41]), biological [42], and microbial [43] degradation and that occurs under subcritical water [44], which has been well summarized by Mueller and Yingling [45], a variety of porous sorbents such as carbon-based materials, ion-exchange resins, or polycationic gels have been investigated to remove PFASs [46,47,48,49]. The adsorption behaviors of PFASs are dramatically affected by the pH values of environmental conditions, as well as substances of several orders of magnitude higher concentrations than PFASs in natural water sources [46,47,48]. Therefore, selective PFAS separation under environmentally friendly conditions has been revealed to be highly challenging [49,50]. In reality, endowed with their fluorophilicity feature, fluoropolymer nanoparticles (FPNPs) (e.g., star polymers with fluorinated nanogel cores and hydrophilic poly(ethylene oxide) arms able to remove PFOA from 10 ppm to ppb levels [51,52]) are interesting candidates for PFAS adsorption because they display strong and selective affinity to PFASs. FPNPs, being highly resistant to non-fluorinated ions, are able to spread in the environment.

**Figure 4:**
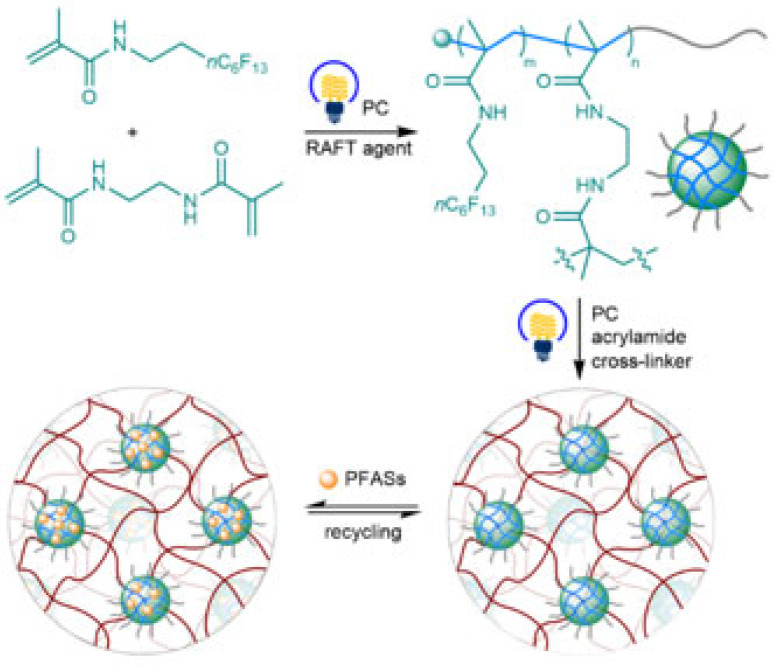
Synthesis of fluoropolymer nanoparticle (FPNP)-embedded hydrogel and study on PFAS adsorption. Adapted with permission from Ref. [54]. Copyright 2020 American Chemical Society.

In addition, Song et al. [53] recently succeeded in eliminating PFOA via chlorine-radical-triggered electrochemical oxidation, while Chen et al. [54] synthesized an original FPNP-embedded hydrogel for PFAS adsorption. These FPNPs were produced from tandem photo-mediated RDRP (Figure 4). The FPNPs showed significant adsorption performance for many cationic, anionic, neutral, and zwitterionic PFASs. The strong and selective affinity toward fluorinated compounds enabled clearance of PFASs at highly environmentally relevant concentrations in water without being dramatically disturbed by acidic/basic conditions. Furthermore, such FPNP-embedded hydrogel displayed good mechanical properties inclined to facilitate its separation, regeneration, and recycling.

Beyond FPNPs, other fluorinated sorbents have been reported as relevant host molecules for PFAS guests. One example deals with cross-linked fluoropolymer gels [46,49], while β-cyclodextrin-based polymers [55] and granular activated carbon [41] are evidenced to capture neutral and/or anionic PFASs efficiently. The outstanding performance of these FPs further highlights that the fluorophilicity effect could be used as a basic feature to promote PFAS adsorption toward a sustainable society.

### 4.2. Fluorinated Polymers

#### 4.2.1. FPs Containing Fluorinated Side Chain or Oxygen Atoms

A wide variety of fluorinated polymers have been reported in some monographs [56,57,58,59] and reviews [60,61,62,63,64]. While fitting the PFAS definition because of their chemical composition, structure, and presence of fluorine atoms [4,7,64], FPs represent a specific family and are significantly different from other substances of the PFAS category (Figure 3). Indeed, they can be composed of (i) polymers bearing fluorinated dangling chains such as poly[fluoro(meth)acrylate]s [65,66], or fluorinated polyurethanes or poly[fluoro(oxetane)]s; (ii) perfluoropolyethers, PFPEs, prepared via ring-opening polymerization of either hexafluoropropylene oxide (HFPO) or oxetanes, or achieved from the photochemical polymerization of TFE and hexafluoropropylene (HFP) in the presence of oxygen [67]; and finally (iii) polymers containing carbon and fluorine atoms in their backbones. As an example, from durable water repellent (DWR) clothing, poly[fluoro(meth)acrylate]s [66] undergo some leaching out of the fluorinated side groups through weathering yielding, PFASs (mainly fluorinated hydroxy telomers).

Furthermore, the microbial degradation of polymers bearing (per)fluorinated side groups represents a major issue, as evidenced by Liu and Avendaño [43]. It can be assumed that, because of their high chemical and ageing stability, to the best of our knowledge, no similar study has yet been reported on FPs bearing fluorine atoms in the backbone. These poly(fluoroacrylate)s possessing ω-perfluorinated dangling moieties have led to 8:2 fluorotelomer derivatives (Scheme 1), while several studies have described the decomposition and release of the fluorinated side chain under various conditions [66].

#### 4.2.2. Fluoropolymers (i.e., Backbone Containing C-F Bonds)

##### Valuable Fluoropolymers with Exceptional Properties

**Figure 5:**
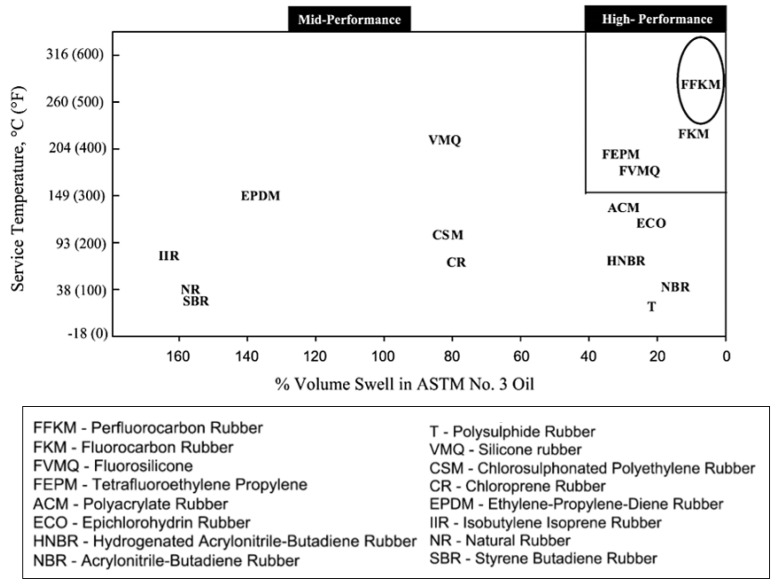
Compared relative heat and oil resistances of elastomers (ASTM D2000). Adapted with permission from Ref. [69]. Copyright 2003 Elsevier.

This sub-section is devoted to polymers bearing C-F bonds within the backbone, and it is divided into four parts. These unique specialty polymers are niche products (production: 330,000 t/year [64] compared to >400 million t/year of all polymers), known to be safe, bioinert, persistent, and not degradable under environmental conditions. These durable materials display outstanding properties [56,57,58,59,60,61,62,63,64], prolonged lifetimes, increased fire safety, reduced maintenance needs, and good performances (even in extreme conditions or aggressive/corrosive media) where other materials fail. One example is the dramatic Challenger explosion in 1986, 73 s after liftoff, related to poly(thioether) seals that had too-high Tg values. Since then, all shuttle packings and gaskets have been made of fluoroelastomers [68]. Advantageously, they are the unique candidates displaying the best heat and oil resistance (e.g., aircraft fluids such as Skydrol^®^) [69] (Figure 5) and excellent flexibility. Essential for our daily life [63] and involved in many high-tech areas, FPs are endowed with exceptional features [56,57,58,59,60,61,62,63,64], with their molar masses being able to reach several million g·mol^−1^ (like PTFE). Because of the growing need for higher requirements and engineering materials that combine specific properties for high-tech applications, FPs are currently undergoing increasing development [70].

One example is poly(vinylidene fluoride), PVDF, which satisfies stringent leachable limits and other national health regulations, ensuring that it is safe for use in water treatment. In addition, this FP is durable, withstanding high pressures while maintaining structural integrity.

Currently, these specialty polymers are also involved in specific UV and aging resistant coatings and have been involved in many other applications: (i) Wires and cables in wide domains such as aerospace (because of their high limiting oxygen index value, PTFE coatings ensure fire retardancy in several-hundred-kilometer cables and connections in planes), insulation (insulation resistance ≥ 1500 MΩ.km), electrical applications, and high-frequency (5000 V) performance in coaxial cables for avionics and high-temperature-resistant cables for aeroengine services [71]. The resilience and inertness of crosslinked elastomers provide exceptional performance to gaskets in shuttles [72]. (ii) Materials for energy storage and conversion (as key candidates for the energetic transition: cathode binders in lithium ion batteries, proton exchange fuel cell membranes, backsheets of photovoltaic panels, electroactive (piezoelectric and electrothermal) devices [73], haptics, printed electronics, dielectrics, for virtual reality, and actuators for artificial muscles, and are indispensable drivers of the European Green Deal). (iii) The chemical process industry (e.g., injection-molded PVDF tower packing is used over and over in sulfuric acid and chlorine scrubber systems and does not lose its properties like other polymers when exposed to harsh chemistries and high temperatures). (iv) Transport (transmission fluids and specific seals and gaskets) [57,74], telecommunications (optical fibers, among more than 4 million km of undersea cables, are carrying information via a huge network) [75], food and water treatment, electronics [76], and pharmaceutical and medical devices [77,78,79,80,81,82].

FPs should only be involved in uses required for health, safety, or when they are critical for the functioning of society and no alternatives are available [9,63,64,83,84]. In addition, searching for alternative materials would reduce the time required for toxicological analyses.

The most prevalent materials are PTFE [61], PVDF [73,85], poly(chlorotrifluoroethylene), PCTFE [86], and the copolymers based on tetrafluoroethylene, TFE [87], and vinylidene fluoride, VDF [88], which can be either thermoplastics or elastomers [72]. Usually, in their conditions of uses, FPs are safe, stable (thermally, chemically against UV and aging, and biologically), durable, non-toxic, non-bioaccumulative, insoluble in water and thus non-mobile, hydrolytically stable, and are not subject to hydrolysis-catalyzed or -metabolized degradation. For these features, they are not considered as substances of very high concern (SVHC) and fulfil the 13 criteria (see Section Polymer-of-Low-Concern Criteria) [35,89,90]. Furthermore, their relevant combinations of properties are not matched by any of the alternative hydrocarbon polymers [9,63,83,84], hence making them so unique and valuable.

Regulatory agencies, such as Registration, Evaluation, Authorisation and Restriction of Chemicals (REACH) [32], the Toxic Substances Control Act (TSCA), and the Japanese Existing New Chemical Substances Inventory (ENCSI), support initiatives aimed at regulating the substances of greatest concern. They will endeavor to contribute to the broad and complex scientific, technical, but also economic forthcoming debate on those substances. Currently, FPs seem to be impacted by the upcoming restrictions on PFASs under the EU REACH Regulation, published in February 2023 [32].

##### Improvement in FP Production in More Environmentally Friendly Processes

Because they are concerned about the negative aspects of the fluorinated polymerization aids (FPAs or surfactants) currently used to replace PFOA, FP manufacturers have been overcoming the great challenge to produce FPs free from FPAs. They are currently searching for solutions for both replacing such PFAs with non-FPAs (NFPAs) and processing by reducing fluorinated effluents and gas emissions. Indeed, a few years ago, the companies Chemours and Dyneon developed and used Gen^®^ex and Adona^®^, respectively, though these FPAs have been found as traces in rivers and soils [34]. However, the regulatory agencies are currently pushed by various authorities towards helping the industry to swiftly move towards sustainable technologies, and many efforts from these FP producers have been recently taken two main strategies: (i) The absence of FPA as well as matching the PLC criteria are more appropriate to specific syntheses (such as FKM). A lot of progress has been observed in the last decade after several FP manufacturers modified their production using NFPAs (or surfactants), leading to major innovations in the whole industry (such as 3M [91], AGC [92], Arkema [93], Chemours [94,95], Daikin [96], Gujarat Fluorochemicals [97,98,99], Solvay [100], and others), with multiple patents being filed claiming such “NFPA Technology” for various products (Table 1) [101].

**Figure 6:**
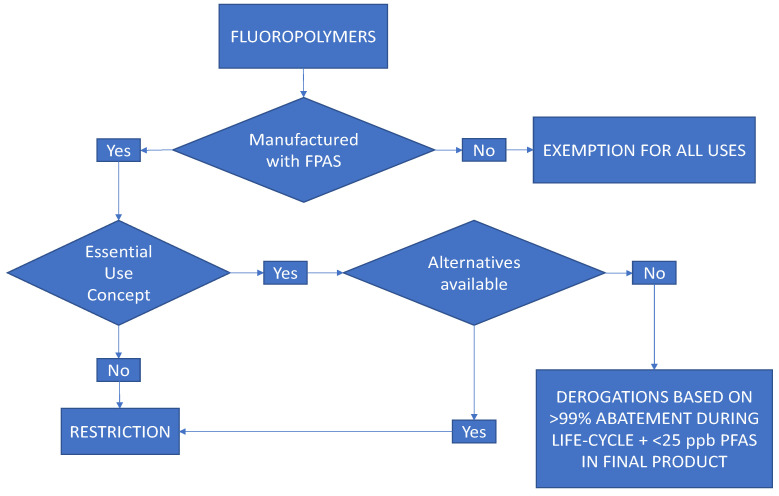
Regulatory decision tree for fluoropolymers. Reprinted from Ref. [101].

However, the use of NFPAs in a specific recipe (for example, in the aqueous radical polymerization of M1 fluoromonomers, e.g., TFE) does not necessary lead to the same success as that of another M2 monomer (e.g., VDF or a mixture of various fluorinated comonomers). The legislation process should be focused on the use of FPAs and the emissions of fluorinated derivatives. In this regard, a regulatory decision tree was suggested [101] (Figure 6), linked to the manufacturing of FPs in the presence or absence of such surfactants (with process abatement to obtain a PFAS concentration lower than 25 ppb [38]), to provide essential use criteria in terms of safety, performance, health, improvements in the process, and alternatives.

Hence, FPs produced without any FPAs should be exempt for all uses across all industries including consumer applications as they raise no risk to the environment or to mammal and human health, in addition to the fact that FPs also match the PLC criteria.

(ii) Manufacturing modification. The second action taken by many FP manufacturers aims at changing their process to eliminate fluorinated side-products such as residual reactants, oligomers, low-molar-mass derivatives and intermediates, aqueous residues, and volatiles. Actually, improving the abatement processes and PFAS recovery in the manufacture of FPs has enabled a reduction of 99% in fluorosurfactant emissions since the 1990s [102], while recent studies have brought it as high as 99.99% [103]. For example, though critical analyses were published in the last decade [104], the Arkema Company strongly reacted and announced that, since February 2023, this innovative solution has made it possible to reduce C_6_F_13_-telomer sulfonate (6:2 FTS) emissions by more than 90% and that the emissions of such a telomer now represent less than 1 kg per day [105] in FP manufacturing in Pierre-Bénite (France) without the use of any fluorosurfactants. Actually, by the end of 2024, a similar process will equip all its other production sites around the world [105].

Other companies have also been modifying their process to significantly reduce their emissions, with many of them having announced such strategies [105,106,107,108,109,110,111,112]. An abundant body of literature is available in relation to the control and treatment of emitted PFASs from industrial processes [43,44,45], while many studies are still ongoing [39,113].

##### Polymer-of-Low-Concern Criteria

More recently, the American Chemical Council led the Fluoropolymer Industry Group (*Fluoropolymergroup*) to investigate a similar study on 14 additional fluorinated (co)polymers [90]. FPs and the Stockholm Convention persistent organic pollutants (POPs) criteria meet the persistence criterion only, but not the bioaccumulative, toxic, or mobile criteria.

According to the OECD definition [1,36,114], the criteria for polymers of low concern (PLC) that result from the combined experience and knowledge of global regulatory authorities on predictive parameters of health and environmental hazards induced by polymers have been extensively reported in several reviews [114,115,116]. Indeed, to be regarded as a PLC, a polymer must not have any known hazard classification while it should also commit to the following criteria:

(i) A high average molar mass number (*M*_n_) and a minimum oligomer amount are the most important requirements for PLC assessment, as claimed by the EU: [115] “*most potential health concern polymers have a low number average molecular weight, M_n_, (<1000 Da) and an oligomer content >1%*.” The higher the number of oligomers, the more eco-toxicological the polymer is [114]. By comparing the potential health concern of polymers with varying oligomer percentages, “*the distribution of potential health concern polymers showed an increased incidence of higher oligomer content that began at 5% for <1000 Da and 2% for <500 Da oligomeric content*” [114];

(ii) Reactive functional group (RFG) requirements;

(iii) Solubility in solvents and in water lower than 10 mg·L^−1^;

(iv) Other criteria such as a low cationic density, not containing any CF_2_ or CF_3_ groups, being stable under the conditions in which it is used. The primary concerns for such FPs are their degradation in the environment to release persistent, bioaccumulative, or toxic products, not being a high-molar-mass water-absorbent polymer (≥10,000 g·mol^−1^), and having any known hazard classification.

Indeed, regarding the RFGs and RFG ratio to *M*_n_ [90,115,116], FPs fulfill the PLC criteria. A PTFE most typically bearing terminal –CF_3_ groups [36] is not considered as an RFG. Furthermore, the *M*_n_ value is an important feature of biological effect because very large molecules (>1000–10,000 Da) are too big to cross the cellular membrane [117,118,119] and thus cannot react with “target organs”, such as the reproductive system. Hence, as the *M*_n_ of a polymer increases, a reduced incidence of potential health concern effects might be expected [114], and, as proof, expended PTFE (e-PTFE) materials are currently used for stents, cardiovascular prostheses, and many other medical items [80,81].

In contrast and as expected, a polymer does not meet the PLC criteria if it decomposes considerably, or degrades or depolymerizes during use. In other words, it undergoes a deep modification into simpler, lower-molar-mass chemicals as the result of oxidation, hydrolysis, heat, sunlight, attack by solvents or microbial action, or any other reaction(s). Indeed, FPs are resistant to such reactions/decompositions [59]. Based on such relevant features and the above PLC criteria, Henry et al. [35] supplied key evidence on four main FPs (PTFE, FEP, PFA and ETFE) matching the 13 PLC criteria [120]. Using gas chromatography/mass spectrometry (GC/MS) and liquid chromatography/mass spectrometry (LC/MS) analyses, these authors characterized the extractible fractions from these FPs and detected that less than 2 ppm was leachable from PTFE (Table 2) [35]. As expected, since the monomers are gases, they were not detected.

Advantageously, as with most FPs, PTFE is insoluble in water and, therefore, is not mobile in the environment. Using the descriptive solubility table from the US Pharmacopeia [121], the water solubility of PTFE would be classified as insoluble (10^–5^ mg·L^−1^ or 0.01 mg·L^−1^) to very slightly soluble (10^–4^ mg·L^−1^ or 0.1 mg·L^−1^).

The polymer policies of 10 countries, including the EU REACH handling of polymers, were examined [114] and it was concluded that “Polymers with <1% of *M*_n_ < 1000 Da and low water extractivity are not able to cause systemic effects which are toxicologically or ecotoxicologically relevant”. In the case of high-molar-mass FPs, such characteristics are not observed (Table 2).

Among all FPs, PTFE is not a substance currently registered under REACH regulations supplying the definition of a polymer substance: “*a molecule that contains a sequence of at least 3 monomer units, which are covalently bound to at least one other monomer unit or other reactant*” [122]. But, because PTFE, like all FPs, is an identifiable organic substance, the suggested Universal Basic Asset (UBA) framework for assessment using the proposed PMT criteria (persistent, mobile, and toxic) would be applicable. Moreover, PTFE is highly stable in the environment and is resistant to thermal degradation. It is stable for years at temperatures up to 260 °C [36,123,124], it is stable to hydrolysis, oxidation, and light, as well as to anaerobic and aerobic degradation [125]. Therefore, PTFE would fulfill the UBA’s proposed persistence criterion.

As stated by USEPA, regarding PFASs and PFCA, the regulatory agency suggests a clarification about the nature of the linkage, stating “*How these materials are incorporated into the polymer is immaterial (they may be counter ions, terminal/end capping agents, or part of the polymer backbone*)” [126]. Surprisingly, the key characteristic is the presence of a –CF_3_ group that is attached to, or forms part of, the polymer backbone and “*this link (between the polymer backbone and the –CF_3_ group) is susceptible to degradation and cleavage*” [126]. Thus, based on a USEPA report, the presence of the –CF_3_ group is relevant since it provides a structural alert to consider potential degradation products [127]. This statement is quite surprising since a CF_3_ end-group prevents depolymerization via unzipping [59].

As listed in Table 2, these FPs are not subject to degradation.

Another advantage of PTFE is its non-toxicity. Thanks to the too-high (several million) average *M*_n_ of PTFE mentioned above, it cannot cross the cellular membrane, indicating it is not toxic or bioavailable. Indeed, this polymer has been extensively tested in the European Union and USA to allow commercial applications for global medical device regulations, food contact, and surgery [77,78,79,80,81]. Furthermore, FPs contain none or tiny monomer(s) (which are gaseous), oligomer(s), and leachable amounts of or no reactive functional groups with high toxicity (Table 2) [36]. These comparisons of PLC and various regulatory assessment criteria demonstrate that, in contrast to conventional PFASs, high-molar-mass FPs display quite different characteristics. Therefore, they should fall into a separate class of materials that must be determined on their own features.

More recently, the American Chemical Council led the Fluoropolymer Industry Group (*Fluoropolymergroup*) to investigate a similar study on 14 additional fluorinated (co)polymers [90]. FPs and the Stockholm Convention persistent organic pollutants (POPs) criteria meet the persistence criterion only, but not the bioaccumulative, toxic, or mobile criteria. Moreover, their physico-chemical properties prevent bioavailability, bioaccumulation, toxicity, and degradation. Presently, 96% of FPs fulfill these 13 PLC criteria.

##### Recycling, End of Life, and Reuse of FPs

Recycling is the re-introduction of utilized compounds (or polymers) into the cycle of products (i.e., polymers in the context). They are collected, sorted, and refined to be re-used as materials or energy source. Recycling should help to preserve resources and to avoid waste [128,129,130]. Indeed, the recycling of plastics is a real challenge [131,132,133] since only ca. 9% of polymers is recycled, which is just under three times more than FPs [134]. The global plastics production was 460 million t (Mt) in 2021 [131], and is predicted to reach 1120 Mt annually by 2050 [132], and should increase up to 1231 Mt by 2060. In the 2000–2019 period, the growth of plastics has outpaced that of economic growth by almost 40%. MacLeod et al. [133] highlighted the global threat induced by plastic pollution based on the high environmental persistence of plastics. In contrast to commodity polymers, the consumption of FPs represents quite a small volume (estimated at 330,300 metric t in 2021 [64], hence representing less than 0.1% among all polymers). In 2015, its global consumption was 270 kt, evidencing a 22.2% increase since then. Actually, the global situation of the fate of FP waste is as follows: 83.5% is incinerated, 13.1% goes to landfill, and 3.4% is recycled [134,135]; this is briefly summarized hereafter.

(i) Different techniques of recycling.

FP waste from commercially available and industrial waste manufacturers is either pre-sorted or arises from dismantling processing, and can also be incinerated for energy recovery. Fractions of pre-sorted FP waste are devoted to recycling, either to be exported for recycling in various countries [134,135,136] or to domestic recyclers. Other forms of recycling include sintering, re-grinding, or chemical recycling of FP materials. Four main methods of recycling are possible: [128] (i) primary (or mechanical) recycling; (ii) secondary recycling; (iii) tertiary recycling; and (iv) quaternary (or energy) recycling.

(ii) Decomposition and recycling of FPs

**Scheme 2:**
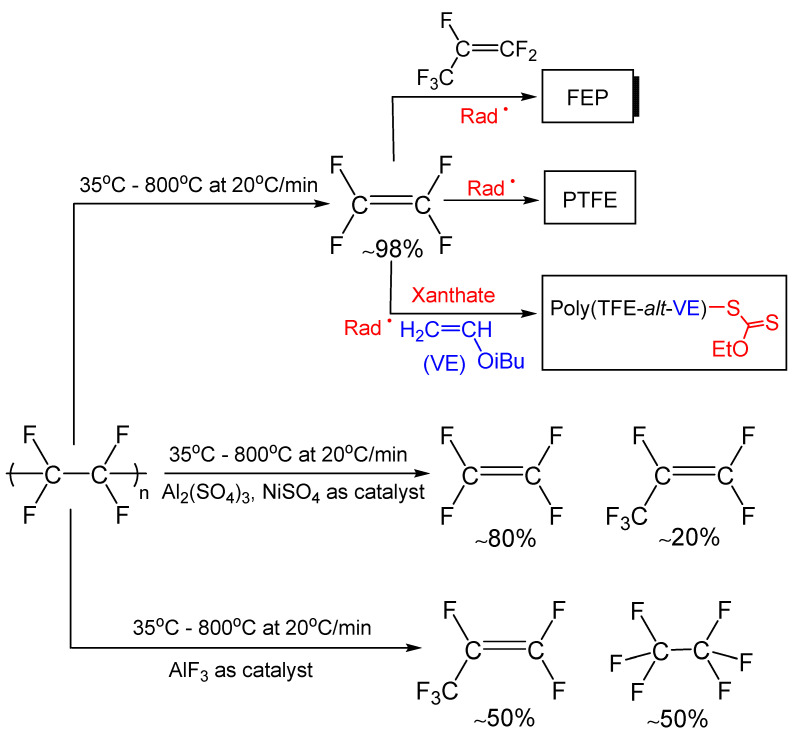
Thermal decomposition of PTFE in absence or presence of various metals or salts to lead back to TFE (and other fluorinated compounds) [142,143]. The recovered TFE was homopolymerized [157], or copolymerized with HFP [158] or isobutyl vinyl ether (iBuVE) [159], to produce PTFE, poly(TFE-*co*-HFP) copolymer (i.e., FEP), or alternated poly(TFE-*co*-iBuVE) copolymer [159].

Several examples are supplied hereafter:

*PTFE*. The thermal degradation and pyrolysis of PTFE was studied by many authors. In 1947, it was pioneered by Lewis and Naylor (under vacuum at 600 °C) [137], 9 years later by Wall and Michaelson (at 450–510 °C, under the presence of various gases) [138], then reported by Simon and Kaminski [139] (who pyrolyzed PTFE at 500–600 °C in a fluidized bed reactor, with the primary products of decomposition being TFE and ^•^CF_2_^•^ bisradicals), comprehensively described by Ellis et al. [140], followed by Schlipf and Schwalm [141], further updated by Puts and Crouse [142,143], and then carefully reviewed by Lakshman and Chakraborty in 2015 in a book [144]. Puts and Crouse [142,143] were able to deeply detect and quantify the released fluorinated compounds (especially fluoroolefins and octafluorocyclobutane, OFCB) in the pyrolysis of PTFE from 35 °C to 800 °C in the presence or absence of various metals or salts. These authors highlighted that, in the absence of salts, TFE was produced in 98% while the nature of the salt induced the release of other gases with the influence of inorganic oxides of Al, Cr, Co, Cu, Fe, Ga, In, La, Mn, Ni, V, Zn, and Zr (Scheme 2) [142,143].

**Figure 7:**
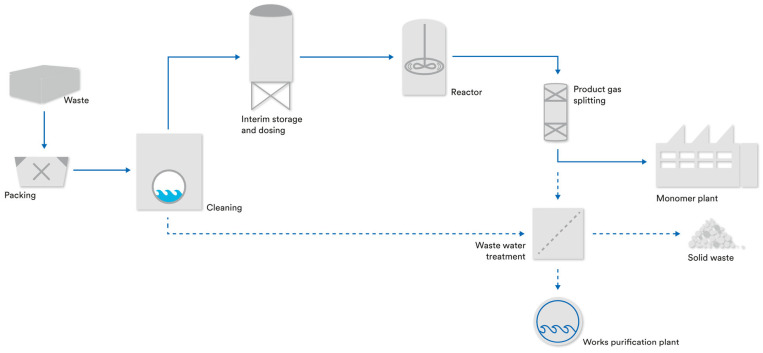
Sketch of the world’s first pilot plant for recycling perfluorinated polymers in Germany. Reprinted with permission from Ref. [145]. Copyright 2016 3M/Dyneon GmbH.

Industrially, while various thermal processes (smoldering, pyrolysis, open burning, etc.) were claimed in the patent literature (recently summarized) [136], the reuse of fluoromonomers has scarcely been reported. However, it was scaled up in a pilot plant at Dyneon Company [145,146] (Figure 7), via a robust process called “*FP upcycling technology*”, yielding more than 90% TFE/HFP. This technology was scaled up into a new demonstration plant (capacity is presently >500 t year).

*Recycling of other thermoplastics*. The company Arkema [141,142] claims to use PVDF copolymers as a processing and recycling aid (PPRA) for polyethylene and polypropylene films, pipes, cables, and injection-molded parts. Such a PPRA increases output and flow, enhances surface finish, reduces extruder pressure, and allows for steady gauge control and processing at lower temperatures. This company has adopted a process that enables only 20–30% of materials to be recycled before losing some features and being reprocessed, and up to 90 or even 100% of recycled materials are required to make such above items [147]. Materials already containing recycled PPRA behave almost the same as a virgin material in which a polymer processing aid (PPA) was added as a master batch in the extruder [148]. PVDF and VDF copolymers are currently assisting the reprocessing of high-volume polyolefins as a PPRA. In addition, Takahara’s group [149] reported that PVDF used for fishing lines could not be degraded via UV radiation and biodegradation and, thus, may be recycled after use.

*Recycling of Fluorinated Elastomers*. The recycling of fluorinated elastomers has been reported in a few studies, which have been well summarized by Schuster et al. [150]. These authors reported only two relevant methods to achieve the recycling of poly(VDF-*ter*-HFP-*ter*-TFE) terpolymers (also named FKM) in an industrially acceptable way: (i) via milling FKM into fine powders to be mixed to virgin FKM and (ii) through the mechanical devulcanization of FKM, followed by successive compounding with virgin rubber. Both techniques give suitable thermostable products by preserving the mechanical features of original FKM. Indeed, these authors’ review mainly cites many patents, while the quoted articles just refer to the degradation and not to de-vulcanization. These authors concluded that recycling FKM is possible and leads to products with competitive properties to those obtained in the presence of virgin FKM. But, as is known, Schuster et al. [150] stated that fluorinated elastomers are usually crosslinked and thus recycling is rather complex, in addition to the fact that they also contain fillers and additives.

**Figure 8:**
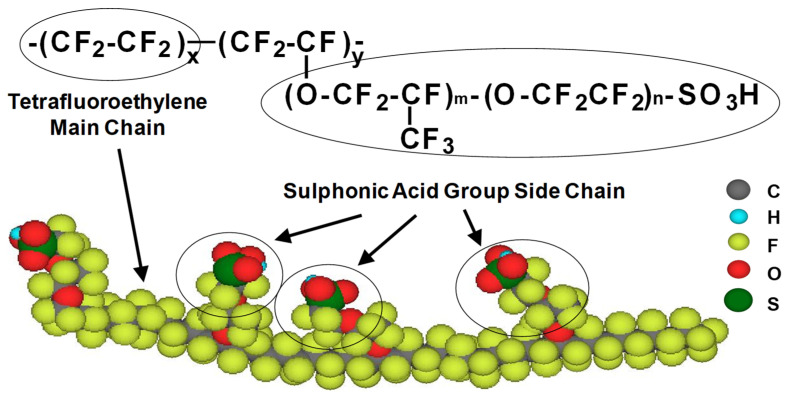
Perfluorosulfonic acid (PFSA) for PEMFC membranes used in fuel cells and electrolyzers. Reprinted with permission from Ref. [154]. Copyright 2016 Royal Society of Chemistry.

*Degradation and recycling of perfluorosulfonic acid membranes.* Another key functional FP is a copolymer based on TFE and a perfluoroalkyl sulfonyl fluoride vinyl ether further processed into proton exchange membranes for fuel cells (PEMFC), such as Nafion^®^, Flemion^®^, Fumion, and Aquivion^®^, called perfluorosulfonic acid (PFSA) membranes (Figure 8) [151]. These high-performance polymers are also involved in chloro-alkali processes to produce chlorine and sodium hydroxide from brine, in desalination for drinkable waters, and in electrolyzers able to produce “clean” hydrogen from water. Though the thermal degradation of Nafion^®^ N117 membranes was reported by Feng et al. [152] and by Zaton et al. [153], its recycling is very challenging.

Nowadays, no alternative is foreseen to be able to substitute such high-performance materials [9,59,63,83,84], which are essential to the functioning of the hydrogen value chain and electrolyzers. These are produced and used in a highly controlled industrial environment, where their emissions are negligible and, due to their high initial price, their reusability and recyclability are actively investigated. So far, a patent from Grot [155] (involving dissolving the membrane and separating the components), a report from Park [156], and a British Research and Innovation project, called Frankenstack [157] (dealing with the recovery and reuse at the end of the lifecycle membrane) are the only documents describing the recycling of PFSA membranes.

##### Chemical Recycling to Monomer and Reuse of the Released Fluoromonomers 

The chemical recycling of FP to fluoromonomers (CRM) is a real challenge. An example is illustrated by Scheme 2 that represents the depolymerization of PTFE into TFE or other fluorinated compounds according to the conditions. The generated TFE could be involved in various reactions enabling the loop to be closed: (i) in radical homopolymerization initiated by peroxides or persulfates to lead to PTFE [158]; (ii) via conventional radical copolymerization of either HFP [159] or isobutyl vinyl ether (iBuVE) [160] to produce either poly(TFE-co-HFP) copolymer (or FEP) or alternated poly(TFE-co-iBuVE) copolymer (as commercially available Zeffle^®^ homologues [154]), and finally (iii) under reversible addition–fragmentation chain transfer (RAFT) copolymerization of TFE and iBuVE, controlled by a xanthate, to yield well-defined poly(TFE-co-iBuVE) copolymers [160].

**Figure 9:**
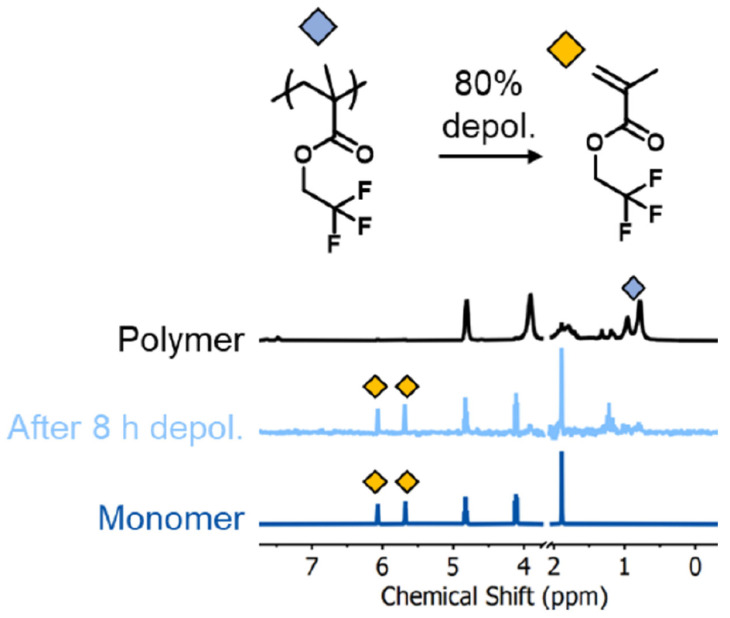
Reaction scheme of the thermal depolymerization of poly(2,2,2-trifluoroethylacrylate), PTFEMA, and ^1^H NMR spectra of PTFEMA (up), of the total product mixture after 8 h (middle spectrum), and of TFEMA after depolymerization (bottom). Adapted from Ref. [161].

Anastasaki et al. [161] reported the catalyst-free depolymerization of poly(2,2,2-trifluoroethylmethacrylate), PTFEMA, at 180 °C. This polymer was previously synthesized via reversible addition–fragmentation chain-transfer polymerization (RAFT) of TFEMA controlled with a trithiocarbonate (TTC). The kinetics of depolymerization, monitored via ^1^H NMR spectroscopy, evidenced the unzipping reaction and evidenced that at the end of the reaction only TFEMA and the TTC remained in the flask (Figure 9). Though it required heat, that reaction did not need any toxic reactants.

##### Reuse of Degraded FP

Bai’s team [162] described that a commercially available FKM elastomer (poly(VDF-co-HFP) or poly(VDF-ter-HFP ter-TFE) fluoroelastomers, Mn > 300,000 g·mol^−1^), the average molar mass in number and dispersity of which were Mn = 134,000 g·mol^−1^ and PDI = 3.7 (while the VDF:HFP molar ratio was 3:1), could undergo tertiary recycling, yielding cooligomers. The Mn of the latter ranged between 3300 and 10,400 g·mol^−1^. This reaction occurred in the presence of sodium hydroxide, hydrogen peroxide, and triethyl ammonium chloride. These transparent dicarboxyl telechelic cooligomers were first chemically changed into diacrylates and then photocrosslinked. The resulting network exhibited suitable mechanical and hydrophobic properties [162].

A more recent work on the use of FEP waste for transparent soils was achieved through the coating of specific terpolymers onto such waste (thanks to fluorine–fluorine interactions). The growing of lettuce roots was monitored using an optical system and the roots were able to live in such an environment [163].

##### Incineration

Incineration is the last method to process end-of-life FPs. Recent studies on the disposal of end-of-life PTFE have highlighted incineration as an appropriate method to dispose of FP too, with no environmental concerns. It is a high-temperature flaming process, which takes place in an open-air space, whereas smoldering is a flameless combustion process that occurs on the surface of a condensed fuel. While both methods can be utilized to decompose fluorinated waste, the main difference is that smoldering is self-sustaining and, hence, is more energy-efficient compared to incineration, which needs a continuous energy input.

Incineration or combustion differ from thermolysis in that a source of fuel is used in order to purposefully favor a complete degradation of FPs (as a source of trifluoroacetic acid or acetate, TFA, and chlorodifluoroacetic acid, CDFA) [140]. Furthermore, due to the high temperatures and oxidizing conditions used, they are unlikely to produce environmentally significant levels of TFA or TFA precursors. On the other hand, the low-temperature burning of domestic waste, as an important source of furans and polychlorinated dioxins to the atmosphere, may analogously be a huge source of fluorinated acids.

Incineration has been adopted by various countries [134,135,164,165]. In the case of Norway [165], though municipal solid waste incineration (MSWI) conditions are achieved at about 850 °C, emissions of FP degradation products from household waste incineration have not been investigated yet. However, at the laboratory scale, the decomposition of PTFE and other FPs was comprehensively studied in the 700–1050 °C temperature range, yielding the release of various main products such as CF_4_, CHF_3_, C_2_F_6_, TFE, HFP, and OFCB (Table 3) [124,140,165,166,167,168,169,170,171].

Most of these substances are non-toxic, but highly toxic ones like perfluoroisobutylene (PFIB) or fluorophosgene (COF_2_) may possibly be released under some severe conditions.

Wang et al. [172] suggested that PFASs may be generated from FP waste via an MSWI process, probably at temperatures lower than 500 °C. Thanks to its high thermal and chemical stabilities, PTFE is inert in the environment and Taylor et al. [173] found that municipal waste incinerators operating with an average gas temperature of 1000 °C can be used to totally decompose PTFE.

**Figure 10:**
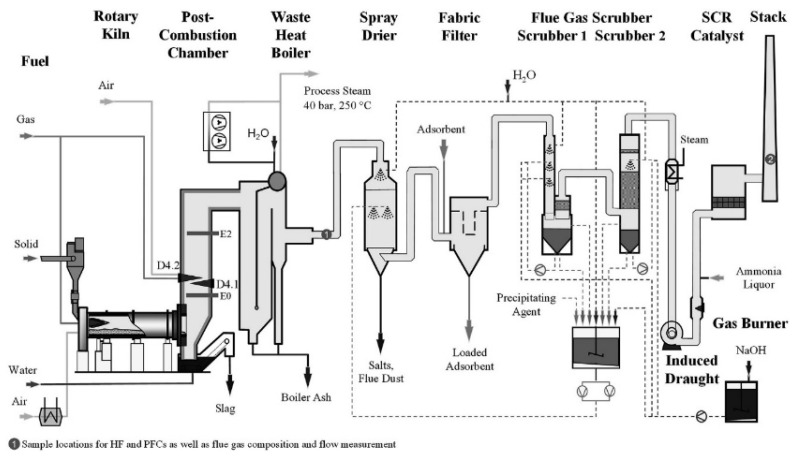
Engineering drawing of the “*Brennkammer mit Dampfkessel*” (BRENDA) (German acronym) pilot combustion facility at Karlsruhe Institute of Technology. Reprinted with permission from Ref. [174]. Copyright 2019 Elsevier.

In contrast, in Germany, Aleksandrov et al. [174] reported that the incineration of PTFE, using the best available techniques (BATs), did not release any PFAS. By comparing with the possible formation of 31 PFAS compounds as references, these authors noted that PTFE could almost fully decomposed into fluorine (as HF) and concluded that the municipal incineration of PTFE should be considered as an acceptable form of waste treatment. They tested for the presence of 31 different PFASs and 11 of these were detected but deemed to be due to contamination from the environment. A constant mass flow of wood pellets was utilized while these authors added PTFE into the reactor to maintain a consistent thermal profile, as shown in Figure 10.

Furthermore, the Dutch Institute for Public Health and Environment (RIVM) [164] drew slightly fewer concrete conclusions, mentioning that although it can be assumed that the polymers are destroyed with the gasification process, this does not provide enough information on the kind and degree of by-products formed or on the rate of mineralization.

**Figure 11:**
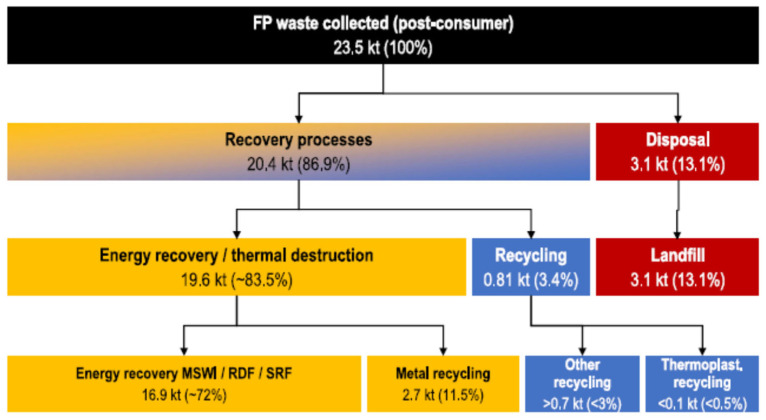
Fate of collected FP waste and tonnages (percentages) involved in recycling, recovery, reuse, and landfill in EU. Reprinted with permission from Ref. [134]. Copyright 2020 Conversio Market & Strategy GmbH.

In the EU, the overall situation of the fate of collected FP waste involved in recycling, recovery, reuse, and landfill, proposed in the Conversio Report, is illustrated in Figure 11 [134,135], indicating that, globally, ca. 23.5 kt of FP waste was collected in 2020 (<0.01% of the total waste). To compare, ca. 29,450 kt of plastics was gathered in 2020 (<5% of the total collected waste excluding mineral fractions). Moreover, about 84% (20.4 kt) of the total FP collected waste in Europe in 2020 is either thermally destructed or (co-)incinerated, while 3.1 kt (13.1%) went to landfill. Finally, 0.81 kt was gathered separately for recycling, whereas a significant amount was exported for recycling (e.g., to Asian countries) [130].

Beside incineration, a more severe degradation of FPs can be obtained through various methods including smoldering and pyrolysis. These are efficient to treat fluorinated waste [136], with the first one being self-sustaining. As a high-temperature process used for thermal decomposition, pyrolysis is similar to incineration except it is achieved in an inert atmosphere. For efficient degradation, a temperature greater than 900 °C is preferred. These techniques enable a total destruction of released hazardous PFASs. Several studies have tested the efficiency of these methods to determine if they reach temperatures high enough for a sufficient duration to enable complete degradation [174,175]. Other techniques of incineration [136], such as the mineralization (vide infra) of FPs during thermal treatment, or a plasma-based water treatment, are also being investigated as they may be safer and more efficient to treat waste.

##### End of Life

Several authors have studied the end of life of FPs [136]. When an FP has fulfilled its intended use and will be disposed of, the fate of the FP must be investigated.

Many reports have concluded that FPs such as PTFE do not degrade in the environment and do not release any compounds hazardous toward mammals, human beings, and the environment [102,123,165,176,177]. Thus, the downstream, end-of-life process of incineration should lead to more studies.

**Figure 12:**
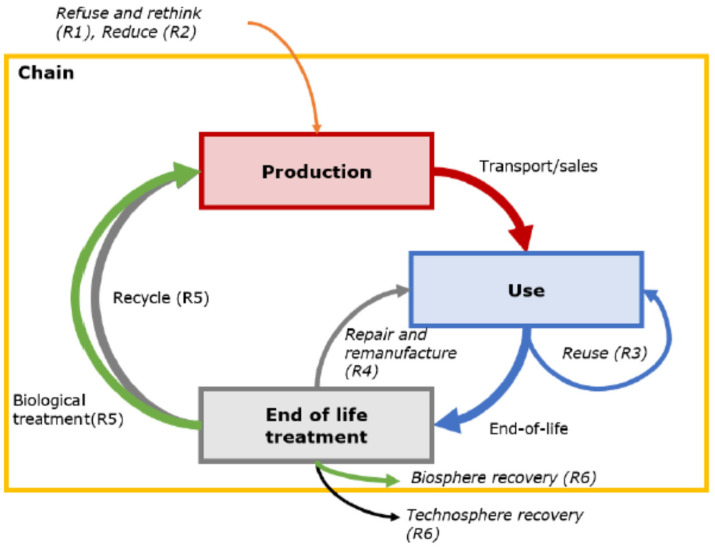
Sketch of flows of materials in a circular economy. Reprinted with permission from Ref. [165]. Copyright 2021 European Topic Centre Waste and Materials in a Green Economy.

In addition, though FPs match the PLC criteria (Section Polymer-of-Low-Concern Criteria), the circular economy of FPs deserves more interest, and Wahlström et al. [165] have comprehensively proposed an overall approach including several flows (Figure 12) ranging from the manufacture of FPs to their use and their recycling (also analyzing the depolymerization of PTFE into TFE as explained in the sections above).

**Figure 13:**
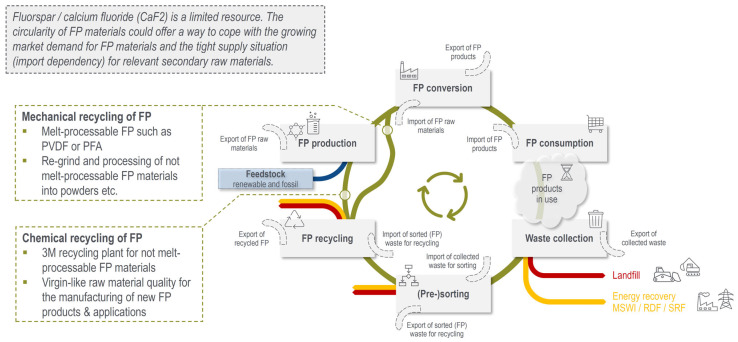
Circular economy situation of FPs: lifecycle of FPs from feedstocks extraction to end of life in EU. Reprinted with permission from Ref. [134]. Copyright 2020 Conversio Market & Strategy GmbH.

The Conversio report [134] extensively provided the results of the treatment of FP waste in different industry segments (automotive, aerospace, semiconductors, electronics [76], and chemical industries) in the EU in 2020 regarding collected waste, energy recovery, landfill, and recycling, as well as the co-treatment of FP and associated waste streams. Figure 13 represents the overall circular economy situation of FPs in the EU, involving pre-consumer processing losses during the manufacturing of FP products and applications [134,135]. Most FPs are (co-)incinerated in MSWI plants or dedicated hazardous waste incineration plants that treat different wastes from chemical waste producers. Indeed, two main methods of recycling have been used (Figure 13): the mechanical and the chemical ones enabling the recycling of melt-processible FPs (such as PVDF or PFA), non-melt-processible ones (as achieved at Dyneon [145,146] or Karlsruhe Institute of Technology [174]), or virgin raw FPs.

The processing of FP materials such as the machining of PTFE rods and cubes for the manufacturing of various milled and drilled parts results in pre-consumer FP processing losses. Currently, ca. 20–25% of FP producers claim to perform their own internal re-processing steps for their process losses. This is the case for Dyneon [145,146], Arkema [147,148], and other companies. Then, 30–35% report that they send their pre-consumer FP waste to external material re-processing companies while 15% export their pre-consumer wastes to other companies inside the EU. Around 5–10% mention that their FP processing losses are exported outside the EU for re-processing, e.g., to Asian countries [130]. Finally, 5% of them state that they do not have any information on their process losses. These companies usually sub-contract waste companies.

##### Mineralization

A total degradation of FPs is desired to avoid any release of oligomers that impart severe persistent and toxic issues. Mineralization, which yields fluoride anions, appears to be an attractive and environmentally friendly method. As reported for low-molar- mass PFASs [44], the mineralization of FPs has been deeply studied by Hori’s group for more than 10 years by means of subcritical (or superheated) water [178]. These conditions can be reached at the critical temperature (374 °C) and pressure (>22.1 MPa) of water [178,179,180,181,182,183,184] for which this fluid displays low viscosity, high diffusivity, and an ability to hydrolyze and mineralize many organic compounds. Indeed, reactions requiring subcritical water are considered environmentally benign with the goal of recycling the fluorine element. In the case of FPs, using this technique, Hori’s team mineralized PVDF [179,180] (decomposed into F^−^ and CO_2_) as well as the VDF copolymers poly(VDF-*co*-HFP) [180], poly(VDF-*co*-CTFE) [179], poly(VDF-*co*-PMVE) [181], and poly(VDF-*co*-MAF) [182] copolymers (where MAF stands for methacrylic acid) and a poly(VDF-*ter*-HFP-*ter*-TFE) terpolymer [183]. This led to a *quasi*-complete mineralization of PVDF performed at 250 °C as well as for poly(VDF-*co*-HFP) and poly(VDF-*co*-CTFE) copolymers [179,180].

**Scheme 3:**
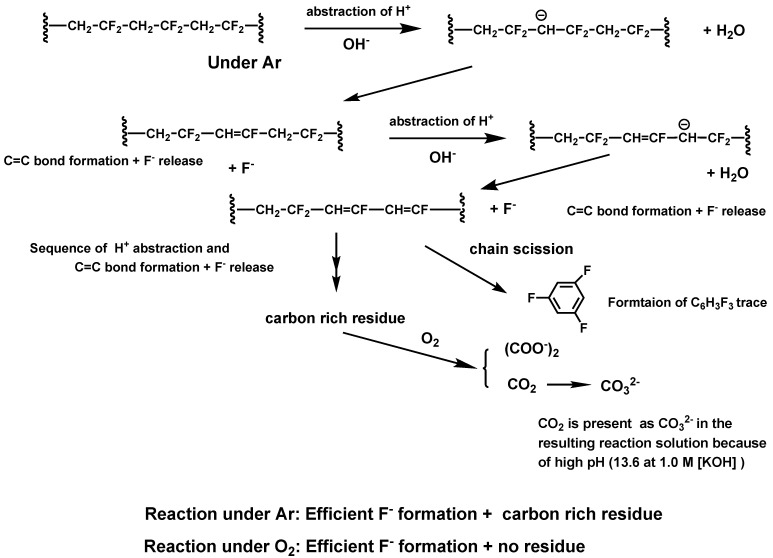
Proposed mechanism for the H_2_O_2_-induced decomposition of PVDF. Reprinted from Ref. [180].

Scheme 3 supplies a suggested mechanism of the decomposition of PVDF. Advantageously, by reacting Ca^2+^ cations (from Ca(OH)_2_) with released fluoride anions, these authors examined that CaF_2_ formation [180,181,182,183] could close the loop on the fluorine element, since CaF_2_ is the source of all fluorinated compounds.

These authors also extended that strategy to the mineralization of poly(VDF-*co*-HFP) and ETFE copolymers [180] and PFSA membranes [184] in much more attractive conditions than those reporting the degradation of PFSA membranes generating perfluoroalkanoic acids [151] and perfluoroalkane sulfonic acids [185].

**Scheme 4:**
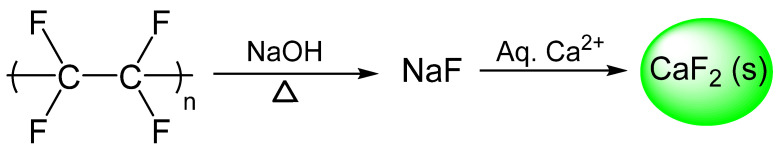
Proposed procedure for the mineralization of PTFE using molten NaOH.

A different approach from another Japanese team [186] involved the use of molten alkaline metal hydroxide at 400–600 °C to mineralize PTFE. This two-step process allowed for efficient mineralization in chemical recycling. However, the process appears surprising since PTFE can be etched by liquid sodium and also degrades from 500 °C, as seen in the Recycling, End of Life and Reuse of FPs section. First, PTFE (as well as PVDF, PCTFE, and poly(VDF-*co*-HFP) copolymer) was decomposed into soluble alkaline fluorides via a degradation in molten hydroxides at high temperatures and atmospheric pressure. Then, CaF_2_, considered the main source of organofluorine chemistry, was produced through treatment of the former aqueous solution with CaCl_2_. When PTFE was heated with a large excess of NaOH at 500 °C, a 74% yield of CaF_2_ was obtained (Scheme 4) with respect to the initial PTFE amount.

Indeed, such high temperatures are not far from the thermal conditions that favor the unzipping depolymerization of PTFE [102,137,138,139,140,141,142,143,144,158] and, consequently, for other less-thermostable FPs than PTFE, degradation should also happen even without any base. These authors noted that (i) there was no decomposition with molten NaOH below 400 °C, whereas (ii) from 600 °C, the yields of CaF_2_ fell down to 46% (from 67% and 74% at 450 and 500 °C, respectively).

**Figure 14:**
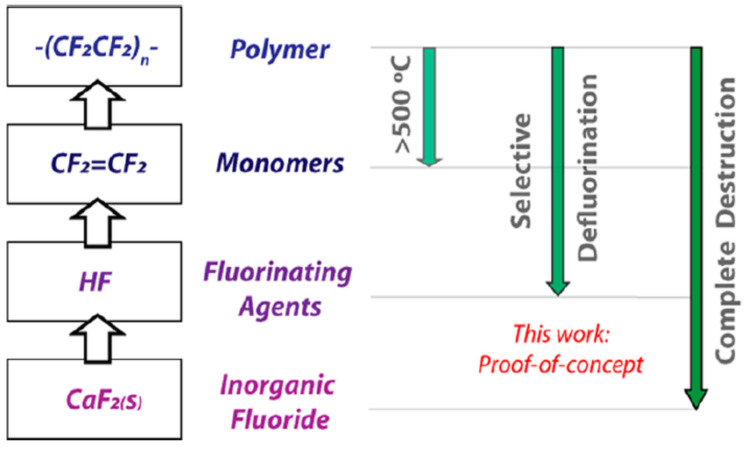
Cycle of fluorine chemistry from fluorspar (CaF_2_) to PTFE, along with potential routes for recycling, including thermal depolymerization (above 500 °C), selective defluorination to form fluorinating agents, and complete destruction back to inorganic metal fluoride. Reprinted from Ref. [187].

**Scheme 5:**
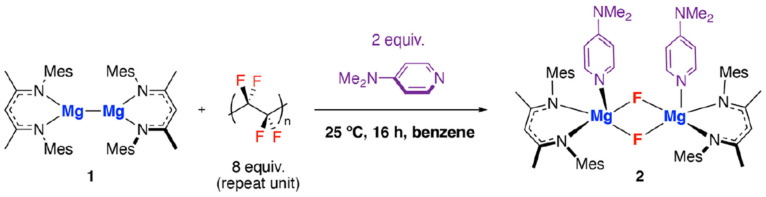
Room-temperature reaction of a nucleophilic magnesium reagent (**1**) with PTFE to generate an organometallic fluorinated magnesium complex (**2**). Reprinted from Ref. [187].

More recently, Sheldon et al. [187] reported that a nucleophilic magnesium reagent (Figure 14 and Scheme 5) could enable the defluorination of PTFE powder under mild conditions (room temperature and 1 atm), releasing a molecular soluble magnesium fluoride coordination product (2) that was separated from the surface-modified FP. This kinetically stabilized species could be utilized as fluoride carrier and appeared more reactive than the inorganic metal fluorides MF(s) or MF_2_(s), which suffer from high lattice enthalpies and high stability. This proof of concept evidences that the fluorine atoms in PTFE can be harvested and reused in coordination synthesis.

## 5. Conclusions and Discussion

PFASs cover more than 12,000 synthetic compounds containing a carbon–fluorine bond. With such a broad scope, not all PFASs are the same and it is likely that the impact of a possible restriction on PFASs on the global industry will be a shocking prospect, particularly since the recent proposal does not differentiate between substances that may have different toxicity profiles and applications. Indeed, the justification to launch a restriction on PFAS as a group is based on the ‘persistency and other concerns’ related to these chemicals and the fact that previous initiatives to regulate PFAS chemicals individually have resulted in the substitution of one PFAS molecule by another molecule that also matches the definition of PFASs, and may exhibit similar toxicological properties.

While most PFASs have not yet undergone toxicology assessments or been linked to health harms, many efforts have been devoted to the detection and remediation in both solid and aqueous media to ensure that contamination is limited to safe levels.

Because of their chemical composition, structure, and much higher molar masses, fluoropolymers are significantly different from other substances in the PFAS family. The only main reason for the concern associated with FPs deals with the use of non-polymeric PFASs as polymerization aids during the manufacturing process (the radical polymerization in an aqueous medium has required a hydrocarbon surfactant to be used for a couple of years).

With such high molar masses (several million for PTFE), FPs cannot migrate through the cellular membrane, hence displaying their non-bioavailability and non-toxicity. Furthermore, due to recent improvements in their manufacturing without any use of fluorinated surfactants, in addition to their high chemical, thermal, and aging stabilities, they contain no or quite tiny oligomer or organic leachables. In addition, their physico-chemical properties prevent bioaccumulation and degradation. Advantageously, as with most FPs, PTFE is insoluble in water and, therefore, is not mobile in the environment. Consequently, the outcomes of such features (in addition the other 13 requirements) are that ca. 96% of the global commercial FPs satisfy the accepted OECD PLC criteria.

In reality, FPs exhibit exceptional properties while preserving their high performance even under aggressive chemical and thermal conditions. These niche technical polymers are stable against hydrolysis, UV, oxidation, and biodegradation. Their applications encompass health, safety, performance, and the functioning of society and they are used in strategic sectors and high-tech industries (e.g., aerospace, electronics, energy, optics, medicine). Their relevant combinations of outstanding features are not observed in any of the alternative hydrocarbon polymers [9,63,83,84], hence making them unique and valuable. Presently, alternatives to FPs, which should be assessed based on criteria such as *performance, safety*, *accessibility*, *resource efficiency*, *waste generation*, *cost*, *availability*, *stability, and lifetime*, do not exist. Furthermore, various reports indicate that FPs (especially PTFE) have been extensively tested to fulfil the Japanese [130], US, and EU food contact and global medical device regulations (e.g., Japan Pharmaceutical and Medical Device Agency, US Food and Drug Administration, Korea Ministry of Food, China Food and Drug Administration, and Drug Safety, including ISO 10,993 for biocompatibility testing and preclinical animal testing).

As presented throughout this review, FPs could potentially present environmental challenges across international borders. Since their inception and widespread commercial and industrial utilizations, these materials have become essential for many everyday applications and are not considered to be a health hazard during their typical life cycle. At their end of life, possible low-molar-mass compounds that are dependent upon certain conditions may become an issue that needs to be easily detected and removed from the environment. Many techniques on the remediation of PFASs have already been achieved.

FPs and the Stockholm Convention persistent organic pollutant (POP) criteria meet the persistence criterion only, but not the bioaccumulative, toxic, or mobile criteria.

Furthermore, the main concerns have been recently addressed, both in using NFPAs and controlling >99% PFAS emissions linked to appropriate abatement technology in industrial FP productions. As a result, FPs produced with NFPA technology have led to commercial success and further motivate the industry to work harder in this direction. In addition, there is a search for various solutions for recycling, reuse, circular economy, and life cycle analysis. In this vein, several Occidental companies have already found strategies to depolymerize, recycle (“close the loop”, even in pilot plants), or reuse FPs. Among the annual plastic production of more than 460 million t [131], only 9% has been recycled while the data fall to 3.4% for FPs (for a yearly production of 330,300 t [64]). Further efforts on the recycling of clean FPs (e.g., PTFE), FEP, PFA, and PVDF waste or scraps generated in production have already achieved success (e.g., by Dyneon for PTFE, FEP, and PFA at a pilot scale [145,146], by Arkema for PVDF [147,148], and at Karlsruhe Institute of Technology [174]); however, the recycling of FPs in consumer articles is not well clarified because they are usually contaminated by several (inorganic) fillers and substances, which hamper that process.

Various studies have shown, on small and pilot (500 t/year [188]) scales, the ability to upcycle FPs back to their monomers (via recycling to monomers) and to use the generated fluoromonomers (e.g., TFE) for further (co)polymerizations. Though such an approach to a closed-loop economy for FPs has already been scaled up, it is expected that additional studies on the recycling and reuse of FPs are currently continuing.

To overcome the limitations of incineration (especially HF release), recent alternative processes regarding the thermal treatment (mineralization to release fluoride anions, precursors of CaF_2_ as the starting point of the fluorine chemistry chain) of FPs either under subcritical water or with molten sodium or a magnesium complex are quite relevant to close the loop and deserve to be further scaled up.

Research is needed to determine the possible emissions from thermal disposal of FPs. Furthermore, methods of thermal degradation and waste treatment of polymers containing PFASs can be greatly expanded on to enable environmentally safe and conscientious processes. In addition, PTFE is known to undergo quite low decomposition above the application temperature of 260 °C for months while 400 °C is required to favor a major degradation. Hence, incineration may potentially degrade FPs, and from 850 °C a total decomposition is observed without releasing any PFAS. Such a growing topic allows efficient degradation temperatures, the potential to release compounds of incomplete combustion, deposition onto land, stack gas analyses, and other risk factors to be evaluated. In addition, reactive extrusion processing has been reported as a versatile technology to recycle melt-processible polymers [189], and could be extended to FPs (except PTFE).

Though many reports evidence that FPs (i) do not degrade in the environment or (ii) do not generate harmful compounds, their incineration requires further innovations. Labelling should comply with eco-labelling standards and thus help EoL managers in identifying and sorting hazardous fluoropolymer waste from other plastic waste during the EoL process.

Though the demand for FPs is still increasing [190], future works should deal with FP manufacturing, highlighting the safety, health, and environmental management practices, under applicable regulations.

FPs could be impacted by the upcoming restriction on PFASs under the EU REACH regulation, the proposal of which was published in February 2023 [33]. A lot of progress has been made in industry over recent years in relation to the control of PFAS emissions due to their use in fluoropolymer production. The FP-industry-representative [191] *FluoroProducts and PFAS for Europe* group (which represents producers, importers, and users of fluorinated products and PFASs across Europe) has issued a six-point action plan, which includes derogations for key applications that are not time-limited.

In addition, the FP industry in Europe [192,193] evaluated the contribution brought by FP manufacturers regarding revenue, investment, and employment, while many more significant benefits can be released along the value chain via the use of FPs in many high-tech applications.

For these reasons and because of their usefulness, inertness, non-toxicity, non-bioaccumulation, and non-mobility, and the growing interest even in upcoming technologies, FPs cannot be in the scope of the PFAS restriction. Moreover, the main concerns linked to the industrial productions of FPs have been addressed (improvement in abatement techniques, removal of PFAS polymerization aids, and introducing alternative technologies). They show that these high-value-added polymers must be separated from conventional PFASs, are essential for everyday life, and are irreplaceable materials.

Hence, the data presented demonstrate that the FP class of PFASs is well defined, safe, and an essential subset of PFASs. Though the dossier is still under evaluation [33] after more than 3000 answers to the consultation, the restriction of FPs under REACH regulations may significantly slow down the EU strategic sustainability ambition [194] as well as the ecological transition. For example, in the hydrogen sector, such a PFAS ban, without granting any exemption, would have catastrophic consequences on the industry’s EUR 30 billion worth of investment in a decade [195] (only including electrolyzers and fuel cells). Such a ban would also jeopardize up to 200,000 direct jobs and over 260,000 indirect ones within 10 years in a market with a potential value of EUR 820 billion that should employ 5.4 million people by the middle of the century.

## Figures and Tables

**Table 1:** Industrial developments of non-fluorinated polymerization aid (NFPA) technology in the manufacturing of fluoropolymers.

Type of Fluoropolymer	References
PTFE Aqueous dispersions	[83,93]
PTFE Fine powders	[90,94]
PVDF	[88,93,95]
FKM	[89,92,95]
PFA	[94]

FKM stands for poly(VDF-*co*-HFP) or poly(VDF-*ter*-HFP-*ter*-TFE) copolymers while PFA means poly(TFE-*co*-perfluoroalkyl vinyl ether) copolymer, respectively.

**Table 2:** Results of gas chromatography/mass spectrometry and liquid chromatography/mass spectrometry analyses of extractible fraction from PTFE to match the PLC criteria (readapted with permission from Wiley) [36].

Final PTFE Results		
Properties of interest	Concentration	PLC Criteria
* % oligomer	Not detected	<2% *w*/*w* (20,000 ppm)
** residual monomers	Not detected	No limit established by OECD, 2009
Low-molar-mass leachables and extractables	^#^ 2 ppm	No limit established by OECD, 2009

* Polymers of potential health concern have an increased incidence of higher oligomer content that began at 5% for <1000 Da and 2% for <500 Da oligomeric content (OECD, 2009). The table lists the lower limit, 2%. ** The data set used by OECD [114] to establish the PLC criteria was insufficient to establish a universal limit for all residual monomers, though residual monomer content was established as a PLC criteria [108]. According to U.S. EPA’s Safer Choice criteria (SCP, 2015), TFE is a residual of concern, which is not allowed to be present in Safer Choice-recognized products at 0.01% or higher. There is no specific limit on residual monomers in the PLC criteria [114]. ^#^ Isopar K, an unavoidable ambient air contaminant adsorbed to PTFE fine powder, was detected at ≤2 ppm.

**Table 3:** FPs and their main decomposition products at various temperatures from the literature. In parenthesis: PFC code [165] (reproduced with permission from Norwegian Institute of Air Research).

Polymer	*T* (°C)	Main Products	References
PTFE	450	COF_2_. HF	[124]
	400–500	TFE, HFP, PFIB	[166]
	500	HFP, TFA	[140]
	530	CF_4_, C_2_F_6_, TFE, HFP, *c*-C_4_F_8_ (*c*-OFCB)	[167]
	550	CF_2_O, C_2_F_6_, CF_3_CFO, C_5_F_4_, CF_3_CF_2_CFO, (CF_2_)_3_O_2_	[168]
	600–700	TFE, OFCB	[169]
	750–800	HFP	[169]
	850–900	PFIB	[169]
	800	CF_4_	[124]
	>900	C_2_F_6_	[124]
	850	HFP, TFE	[170]
	750–1050	C_2_F_6_, CF_4_	[170]
ETFE	350	COF_2_, PFBE, TFE, CO	[124]
ECTFE	500	TFA, CDFA	[124]
FEP	400	COF_2_, HFP, TFE, PFIB	[124]
PFA	400	COF_2_	[124]
PEEPE	500	TFA	[140]
CPTFE/ PCTFE	500	CPFP, CDFA	[124]
PTFE/PFA+ PTFE/FEP	800	CH_4_, CHF_3_ (HFC-23), C_2_F_6_ (PFC-116)	[171]

## Data Availability

Data are provided in the list of references.
